# Thyroid Cancer Central Lymph Node Metastasis Risk Stratification Based on Homogeneous Positioning Deep Learning

**DOI:** 10.34133/research.0432

**Published:** 2024-08-20

**Authors:** Siqiong Yao, Pengcheng Shen, Fang Dai, Luojia Deng, Xiangjun Qiu, Yanna Zhao, Ming Gao, Huan Zhang, Xiangqian Zheng, Xiaoqiang Yu, Hongjing Bao, Maofeng Wang, Yun Wang, Dandan Yi, Xiaolei Wang, Yuening Zhang, Jianfeng Sang, Jian Fei, Weituo Zhang, Biyun Qian, Hui Lu

**Affiliations:** ^1^ State Key Laboratory of Microbial Metabolism, Joint International Research Laboratory of Metabolic and Developmental Sciences, School of Life Sciences and Biotechnology, Shanghai Jiao Tong University, Shanghai 200240, China.; ^2^ SJTU-Yale Joint Center of Biostatistics and Data Science, National Center for Translational Medicine, MoE Key Lab of Artificial Intelligence, AI Institute Shanghai Jiao Tong University, Shanghai 200240, China.; ^3^ Department of Automation, Tsinghua University, Beijing, China.; ^4^ Tongji Hospital, School of Medicine, Tongji University, Shanghai, China.; ^5^ Department of Head and Neck Tumor, Tianjin Medical University Cancer Institute and Hospital, National Clinical Research Center for Cancer, Key Laboratory of Cancer Prevention and Therapy, Tianjin, Tianjin’s Clinical Research Center for Cancer, Tianjin, China.; ^6^ Department of Thyroid and Breast Surgery, Tianjin Union Medical Center, Tianjin, China.; ^7^ Cancer Prevention Center, Tianjin Medical University Cancer Institute and Hospital, National Clinical Research Center for Cancer, Key Laboratory of Cancer Prevention and Therapy, Tianjin, Tianjin’s Clinical Research Center for Cancer, Tianjin, China.; ^8^ Inner Mongolia Xing’an Meng People’s Hospital, Ulanhot, China.; ^9^ Department of Biomedical Sciences Laboratory, Affiliated Dongyang Hospital of Wenzhou Medical University, Dongyang, Zhejiang, China.; ^10^ Department of Oncological Surgery, Xuzhou City Central Hospital, The Affiliated Hospital of the Southeast University Medical School (Xu zhou), The Tumor Research Institute of the Southeast University (Xu zhou), Xuzhou, Jiangsu, China.; ^11^ Division of Thyroid Surgery, Department of General Surgery, Nanjing Drum Tower Hospital, the Affiliated Hospital of Medical School, Nanjing University, Nanjing, 210008, China.; ^12^ Department of General Surgery, Pancreatic Disease Center, Ruijin Hospital, Shanghai Jiao Tong University School of Medicine, Shanghai, China.; ^13^ Department of General Surgery, Ruijin Hospital Lu Wan Branch, Shanghai Jiaotong University School of Medicine, Shanghai, China.; ^14^ State Key Laboratory of Oncogenes and Related Genes, Shanghai, China.; ^15^ Institute of Translational Medicine, Shanghai Jiao Tong University, Shanghai, China.; ^16^ Hong qiao International Institute of Medicine, Shanghai Tong Ren Hospital and Clinical Research Institute, Shanghai Jiao Tong University School of Medicine, Shanghai, China.; ^17^ Shanghai Engineering Research Center for Big Data in Pediatric Precision Medicine, NHC Key Laboratory of Medical Embryogenesis and Developmental Molecular Biology & Shanghai Key Laboratory of Embryo and Reproduction Engineering, Shanghai 200020, China.

## Abstract

Due to the absence of definitive diagnostic criteria, there remains a lack of consensus regarding the risk assessment of central lymph node metastasis (CLNM) and the necessity for prophylactic lymph node surgery in ultrasound-diagnosed thyroid cancer. The localization of thyroid nodules is a recognized predictor of CLNM; however, quantifying this relationship is challenging due to variable measurements. In this study, we developed a differential isomorphism-based alignment method combined with a graph transformer to accurately extract localization and morphological information of thyroid nodules, thereby predicting CLNM. We collected 88,796 ultrasound images from 48,969 patients who underwent central lymph node (CLN) surgery and utilized these images to train our predictive model, ACE-Net. Furthermore, we employed an interpretable methodology to explore the factors influencing CLNM and generated a risk heatmap to visually represent the distribution of CLNM risk across different thyroid regions. ACE-Net demonstrated superior performance in 6 external multicenter tests (AUC = 0.826), surpassing the predictive accuracy of human experts (accuracy = 0.561). The risk heatmap enabled the identification of high-risk areas for CLNM, likely correlating with lymphatic metastatic pathways. Additionally, it was observed that the likelihood of metastasis exceeded 80% when the nodal margin’s minimum distance from the thyroid capsule was less than 1.25 mm. ACE-Net’s capacity to effectively predict CLNM and provide interpretable disease-related insights can importantly reduce unnecessary lymph node dissections by 37.9%, without missing positive cases, thus offering a valuable tool for clinical decision-making.

## Introduction

The global incidence of thyroid cancer has surged by 20% over the past 3 decades, constituting nearly 10% of all reported cancers [1–5]. Central lymph node metastasis (CLNM) is a highlighted concern, afflicting approximately 40% to 70% of individuals diagnosed with thyroid cancer [6–10], and is widely recognized as a prognostic indicator of adverse outcomes [11–14]. However, there is a lack of definitive diagnostic methods and criteria for CLNM. Therefore, a significant controversy surrounds the topic of prophylactic lymph node excision in thyroid cancer surgery. Clinicians grapple with the delicate balance between the potential surgical complications arising from lymph node excision [15–18] and the risk of persistent/recurring malignancies attributed to residual CLNM [19–22]. Guidelines addressing this matter exhibit substantial variability across nations. For instance, Chinese guidelines advocate for prophylactic CLN surgery in clinically node-negative (cN0) papillary thyroid carcinoma (PTC) patients [23–25], while the American Thyroid Association guidelines emphasize the inappropriateness of prophylactic CLN surgery for small (T1 or T2), noninvasive, cN0, and the majority of follicular cancers [26,27]. Therefore, achieving accurate preoperative prediction of CLNM risk, especially through noninvasive ultrasonography, holds the potential to resolve this debate, reduce unnecessary surgical interventions, and confer substantial benefits to individuals diagnosed with thyroid cancer.

Currently, ultrasonography plays a pivotal role in assessing the likelihood of CLNM [19,28,29]. However, it is important to acknowledge that many CLNs are challenging to detect via ultrasound, leading to notable limitations in sensitivity in this approach [30,31]. An alternate strategy involves evaluating the risk of CLNM based on ultrasound characteristics of the thyroid node rather than the lymph node itself. In the existing literature, certain high-risk features associated with CLNM have been identified including large nodule size (>1 cm), presence of microcalcification, multifocal lesions, capsule infiltration, nodule location in the middle and lower thyroid regions, and the minimum distance between the nodule and the thyroid dorsal membrane, among others [28,32–39]. Nodule position was considered the most relevant ultrasonographic feature for CLNM. However, compared to structured data, ultrasound images are more susceptible to variations in the operator’s scanning technique, leading to significant heterogeneity in lesion morphology. Additionally, there is the issue of nonuniform image specifications across multiple resolutions and scales. Furthermore, due to the lack of standardized analysis methods for large-scale data, researchers often rely on subjective measurements and inferences for statistical results regarding nodule position information, leading to inherent difficulties in mitigating reader variability and obtaining reproducible evidence [40–42]. Consequently, previous investigations were constrained to using superficial descriptions, employing conventional metrics such as “inferior region” [43] and “near the dorsal membrane” [44], which resulted in suboptimal predictive performance. Remarkably, these reported risk factors have not yet found inclusion in guideline-recommended decisions [24,27]. Similarly, to the best of our knowledge, there is currently no quantitative CLNM risk score system with documented evidence of strong predictive performance in external validation.

Artificial intelligence (AI) technology is widely applied in the identification of lesions in medical imaging and disease diagnosis, and its performance has reached or exceeded human levels [45–54]. However, its predominant role has been as a predictive tool, with limited capacity in driving medical research and gaining deeper insights into various disease mechanisms. For example, a recent investigation illustrated the potential of deep learning techniques applied to ultrasound images of thyroid nodules, showcasing superior performance compared to human experts in predicting CLNM with an area under the curve (AUC) of 0.93 during external validation [29]. Nevertheless, like the majority of AI models developed in the past, this particular model contributed minimally to novel knowledge available to medical researchers. Consequently, several pressing questions persist: Why does the AI model demonstrate superior performance compared to human experts? What critical insights does AI capture that clinicians may have previously overlooked or disregarded? In light of these inquiries, there exists a significant demand for the development of a novel framework and methodology in interpretable AI, with the explicit aim of elucidating such valuable information.

In this study, we trained and tested the interpretable homogeneous positioning deep learning model named ACE-Net (Ability and Chance Evaluation Network). By integrating the locational information (metastatic chance) and morphological features (metastatic ability) of thyroid nodules in ultrasound images, precise prediction of CLNM was achieved before surgery, providing qualitative and quantitative explanations for the metastatic mechanism. In pursuit of our objectives, we have embarked on pioneered efforts that involve leveraging diffeomorphism technology and a unified template to establish comprehensive distribution statistics for thyroid tumors within the entire population. Furthermore, we have meticulously crafted 2 distinct semantic branches dedicated to morphological characteristics and embedded location data. To elucidate the individual predictive contributions of these factors, we have employed targeted AI interpretation methodologies. Additionally, we have quantified and visually represented the risk of CLNM associated with the location of nodules within the thyroid using a heatmap generated through deep graph convolutional neural networks. To ensure the robustness and generalizability of our findings, we have conducted a nationwide multicenter study, encompassing the development, validation, and external testing of our AI model. Furthermore, we have undertaken an independent study comparing the AI-driven predictions with those made by 105 clinicians, each possessing varying levels of expertise in the field. By adopting AI-based interpretative methodologies ACE-Net not only functions as a valuable predictive tool for CLNM but also offers a novel avenue for gaining insights into CLNM.

## Materials and Methods

### Data collection

We conducted a retrospective analysis by gathering preoperative thyroid ultrasound images from patients undergoing thyroidectomy. Our inclusion criteria were as follows: (a) aged ≥18 years old, (b) must have undergone thyroid ultrasound diagnosis and possessed clear ultrasound images, (c) were subsequently confirmed to have malignant thyroid conditions following thyroidectomy, (d) must have received lymph node dissection and the total number of lymph nodes dissected was ≥5, and (e) with pathologic evaluation for CLNM. Patients were excluded for various reasons, including the absence of pathological reports, thyroid surgery not being the primary procedure, and the receipt of preoperative therapy. Stringent control measures were also implemented to ensure image quality, which entailed excluding cases with unclear image quality, multifocal lesions captured in a single image, and images with measuring lines. In parallel, we collected comprehensive patient demographic data, Kwak Thyroid Imaging Reporting and Data Systems (Kwak-TIRADS) grade [55], postoperative pathology results, and CLNM information as part of our data acquisition process.

**Fig. 1.**
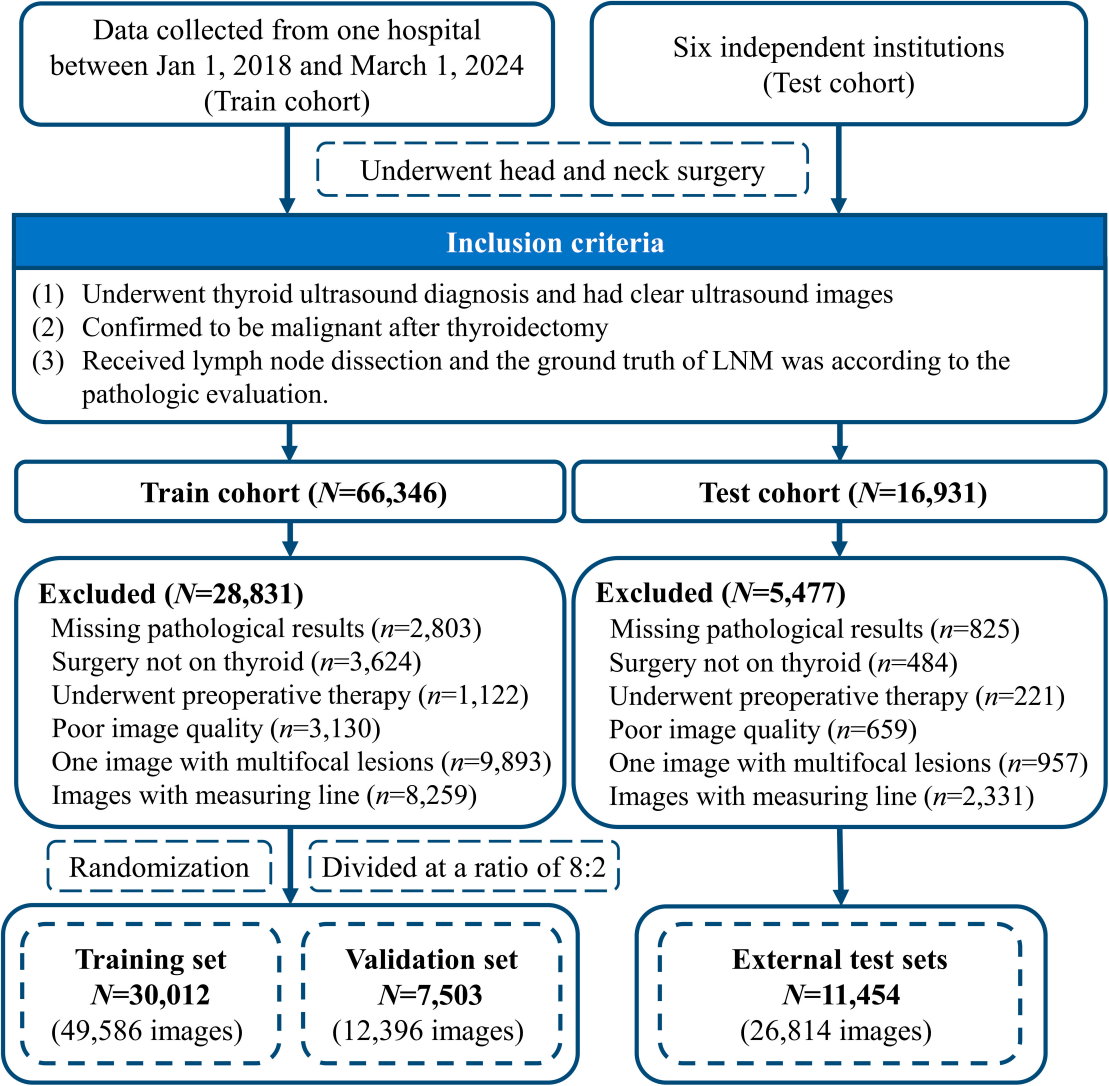
Flow diagram summarizing the inclusion of patients and images. This study consisted of 30,012 patients in the training set, 7,503 patients in the validation set, and 11,454 patients in external test sets.

We gathered ultrasound images from 7 hospitals for train and test cohort between January 2018 and March 2024. Figure 1 shows the patient inclusion and exclusion process. Subsequently, we partitioned train cohort into a training set and validation set, while the external test set comprised patients drawn from 6 independent institutions. This comprehensive approach ensured that our study population represented a diverse cross-section of individuals from various regions and ethnic backgrounds in China. For patient privacy protection, the authors had access to anonymized data only.

To classify patients as CLNM positive, we identified those who had at least one positive lymph node among those removed during surgery. Regarding the meticulous quality control of data annotation, we implemented a 2-step process:

1. The differentiation of malignant nodules: All malignant nodules were diagnosed based on pathological findings. Independent ultrasound physicians, each possessing more than 5 years of experience, were tasked with reevaluating the images. In instances where their evaluation diverged from the original reports, we sought expert judgment to resolve discrepancies.

2. Pathological annotation of CLNM on images: When dealing with patients presenting multiple nodules, ascertaining which nodule had metastasized to the lymph nodes posed a challenge during the annotation process. To address this complexity, we enlisted 3 ultrasound radiologists to meticulously compare the ultrasound images with the corresponding pathological reports for each patient. Their objective was to select the image of the nodule most likely to have metastasized, considering factors such as the nodule’s location and the malignancy.

### The overall framework of ACE-Net

**Fig. 2.**
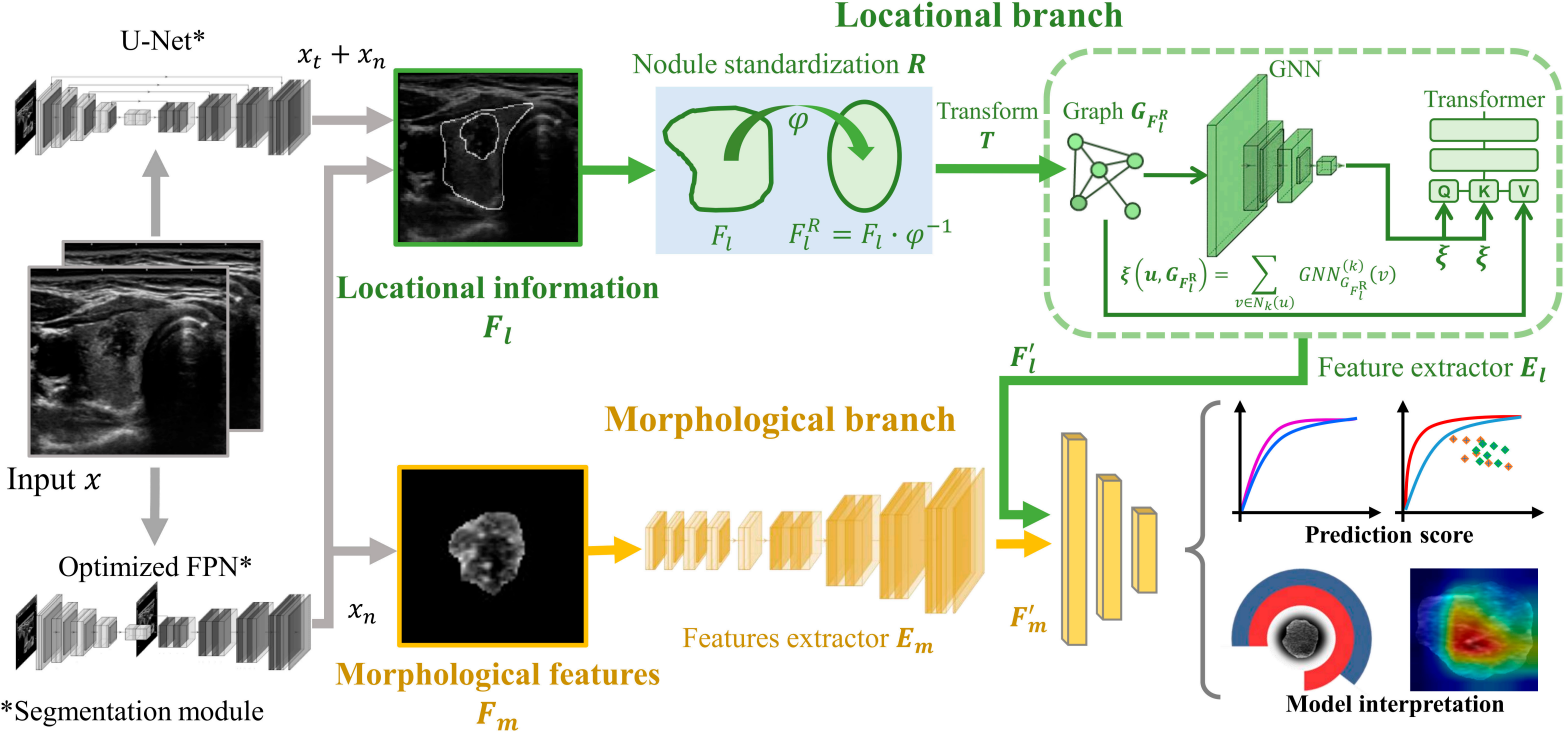
ACE-Net architecture. The input data for the model are an image *x*, which is processed by a segmentation module to delineate the thyroid region *x_t_* and the nodule region *x_n_* within the data. Based on *x_t_* + *x_n_*, the locational information *F_l_* can be computed and input into the locational branch. This branch includes the homogeneous positioning method: *F_l_* undergoes the diffeomorphism *φ* to obtain registered features *F_l_^R^* referred to as the registration process *R*. Then, through transformation *T*, *G_F_* is generated, and representation *ξ* of a node *u* is obtained using a graph neural network (GNN) model, which is input into a transformer structure to extract locational information vectors *F′_l_*. This part constitutes the structure-aware graph transformer (SAT), represented as feature extractor *E_l_* structure. The morphological features *F_m_* of the nodules are obtained from *x_n_*, and morphological feature vectors *F′_m_* are extracted using the feature extractor *E_m_*, which is based on Neighborhood Attention Transformer (NAT) network. These 2 sets of features are integrated into a unified prediction score. To facilitate model interpretation, we used ANOVA to discern the individual contribution of different factors. Additionally, we generated heatmaps depicting the risk of CLNM.

To enhance the accuracy of CLNM prediction and offer comprehensive clinical insights to inform physician decision-making, we devised an advanced deep learning framework named the ACE-Net, which combined nodule morphological features (metastatic Ability) with locational information (metastatic Chance) to predict CLNM. The primary objective was to elucidate the individual contributions of diverse ultrasonographic information types in the prediction of CLNM as illustrated in Fig. 2. This network architecture was designed to feature 2 embedded branches. The locational branch effectively extracts the relative locational information of nodules through the homogeneous positioning method.

We employed the UNet [56,57] and optimized Feature Pyramid Network (FPN) [58,59] networks for the segmentation of nodules and the thyroid boundary. Subsequently, we designed a 2-branch architecture to independently extract morphological and locational characteristics of nodules, combining the prediction results of each feature to derive the final CLNM prediction outcome:

1. Morphological branch: In this branch, we provided the interior area of the segmented nodule as input to the Neighborhood Attention Transformer (NAT) [60] network for predicting CLNM. Consequently, only the morphological attributes of the nodule were utilized, with locational information being masked within this branch.

2. Locational branch: We developed the homogeneous positioning method to establish a standardized 3D positioning of thyroid nodules across a population. In this method, we first conducted structure-enhanced diffeomorphic affine transformations [61,62] on the data for spatial positioning standardization. We then extracted key positional information and transformed it into graph network structured data, utilizing the structure-aware graph transformer (SAT) [63] as the foundational architecture for extracting location features.

In our model interpretation process, we employed analysis of variance (ANOVA) [64–66] techniques to discern the respective contribution of various factors. Additionally, we utilized saliency map [67] to create heatmaps depicting the risk of CLNM based on insights obtained from the SAT. This approach allowed us to construct location relationships in a higher-dimensional space, facilitating a more effective exploration of the inherent correspondences within each piece of locational information. Furthermore, it quantified the pivotal edge points that played a significant role in the prediction process. Consequently, our study introduces a novel methodology that transforms the positional relationship between the thyroid and nodules into a graph structure. See Sections S1 to S5 and Fig. S1 for comprehensive descriptions regarding the complete network architecture.

### Model evaluation and human–AI competing test

The performance of the prediction model was evaluated by assessing the AUC of the receiver operating characteristic curve (ROC), as well as its sensitivity, specificity, negative predictive value (NPV), and positive predictive value (PPV).

To facilitate a comparative analysis between AI and human experts, we engaged clinicians through the public website (https://bmap.sjtu.edu.cn/platform/details/74) to make prediction regarding CLNM based on ultrasound images. All participating clinicians possessed a CDFI technician certification and had reported experience in thyroid ultrasonography. Additionally, each clinician was tasked with 2 specific responsibilities:

1. Task 1: Predict CLNM based on 200 ultrasound images, comprising 100 cases with CLNM and 100 cases without CLNM.

2. Task 2: Diagnose thyroid cancer based on 200 ultrasound images, featuring 100 benign and 100 malignant nodules.

Task 2 was designed to serve as a qualification examination, evaluating the ultrasonographic expertise of the participating clinicians.

Furthermore, we conducted assessments of the predictive performance of features identified by human experts, encompassing “Thyroid Imaging Reporting and Data Systems (TIRADS) score”, “nodule size”, “irregular margins”, “hypoechogenicity”, “taller-than-wide”, “microcalcifications”, “back membrane breakthrough”, “inferior region”, and “upper region”. Notably, the “TIRADS score” exhibited the most favorable performance and was consequently chosen as the baseline reference for evaluating the supplementary predictive capability of the AI model concerning CLNM.

### Ethics

This study was approved by the institutional review board (IRB) of Shanghai Tong Ren Hospital and undertaken according to the Declaration of Helsinki. Informed consent from patients with thyroid cancer and controls was exempted by the IRB because of the retrospective nature of this study.

### Statistical analysis

We estimated the 95% confidence intervals (CIs) solely for the performance metrics pertaining to our classification results through bootstrapping, including AUC, sensitivity, specificity, NPV, and PPV. Our approach involved implementing *n*-out-of-*n* bootstrap sampling with replacement at the image level for our datasets. For each bootstrap sample, we computed and retained the performance metrics specific to that sample. This process was repeated 1,000 times. Subsequently, we derived 95% CI by utilizing the 2.5 and 97.5 percentiles from the empirical distribution of the respective metrics. All computations and statistical analyses were performed in Python, version 3.8 (Python Software Foundation).

## Results

### Data description

We gathered a total of 88,796 ultrasound images from a cohort of 48,969 patients, and the clinical characteristics of our study population are presented in Table. The data on the occurrence and nonoccurrence of CLNM exhibited a high degree of similarity. Notably, there was a 4-fold greater representation of female patients compared to their male counterparts. Furthermore, approximately 94% of the patient cohort was identified as of Han descent. The TIRADS grades were predominantly concentrated in 4A, 4B, and 4C.

**Table.** Characteristics of the patients at baseline^a^

Characteristic	Training set	Validation set	External test sets	Total
Participants, no.	30,012	7,503	11,454	48,969
Cancer participants, no. (%)				
Metastatic	15,106 (50)	3,761 (50)	5,883 (51)	24,750 (51)
Nonmetastatic	14,906 (50)	3,742 (50)	5,571 (49)	24,219 (49)
Median age, years (range)	49 (18–79)	48 (18–79)	50 (18–78)	49 (18–79)
Sex, no. (%)				
Female	25,184 (84)	5,877 (78)	8,542 (75)	39,603 (81)
Male	4,828 (16)	1,626 (22)	2,912 (25)	9,366 (19)
Nation, no. (%)				
Han nationality	29,169 (97)	7,358 (98)	9,605 (84)	46,132 (94)
Minority nationality ^b^	843 (3)	145 (2)	1,849 (16)	2,837 (6)
Kwak TIRADS, no. (%)				
1–3	289 (1)	72 (1)	463 (4)	824 (2)
4 (4A–4C)	26,155 (87)	6,357 (85)	9,088 (79)	41,600 (85)
5–6	3,568 (12)	1,074 (14)	1,903 (17)	6,545 (13)
Histological subtypes				
Papillary thyroid cancer	27,474 (92)	6,871 (92)	10,317 (90)	44,662 (91)
Follicular thyroid cancer	2,056 (7)	512 (7)	834 (7)	3,402 (7)
Medullary thyroid cancer	482 (2)	120 (2)	303 (3)	905 (2)
Metastatic ratio, no. (%) ^c^				
0	14,301 (49)	3,575 (48)	6,132 (54)	24,008 (49)
0–50	5,382 (18)	1,345 (18)	1,707 (15)	8,434 (17)
50–100	10,329 (34)	2,583 (34)	3,615 (32)	16,527 (34)

^a^ Percentages may not add up to 100 due to rounding.

^b^ The ethnic groups listed in the table corresponded to those indicated on the patients’ identification documents for the surgical procedure. Among the represented ethnic minorities were Mongolians, Manchus, and Huis.

^c^ The metastatic ratio represents the proportion of metastatic lymph nodes relative to the total number of lymph nodes removed during the procedure.

### Prediction performance of models and human experts

**Fig. 3.**
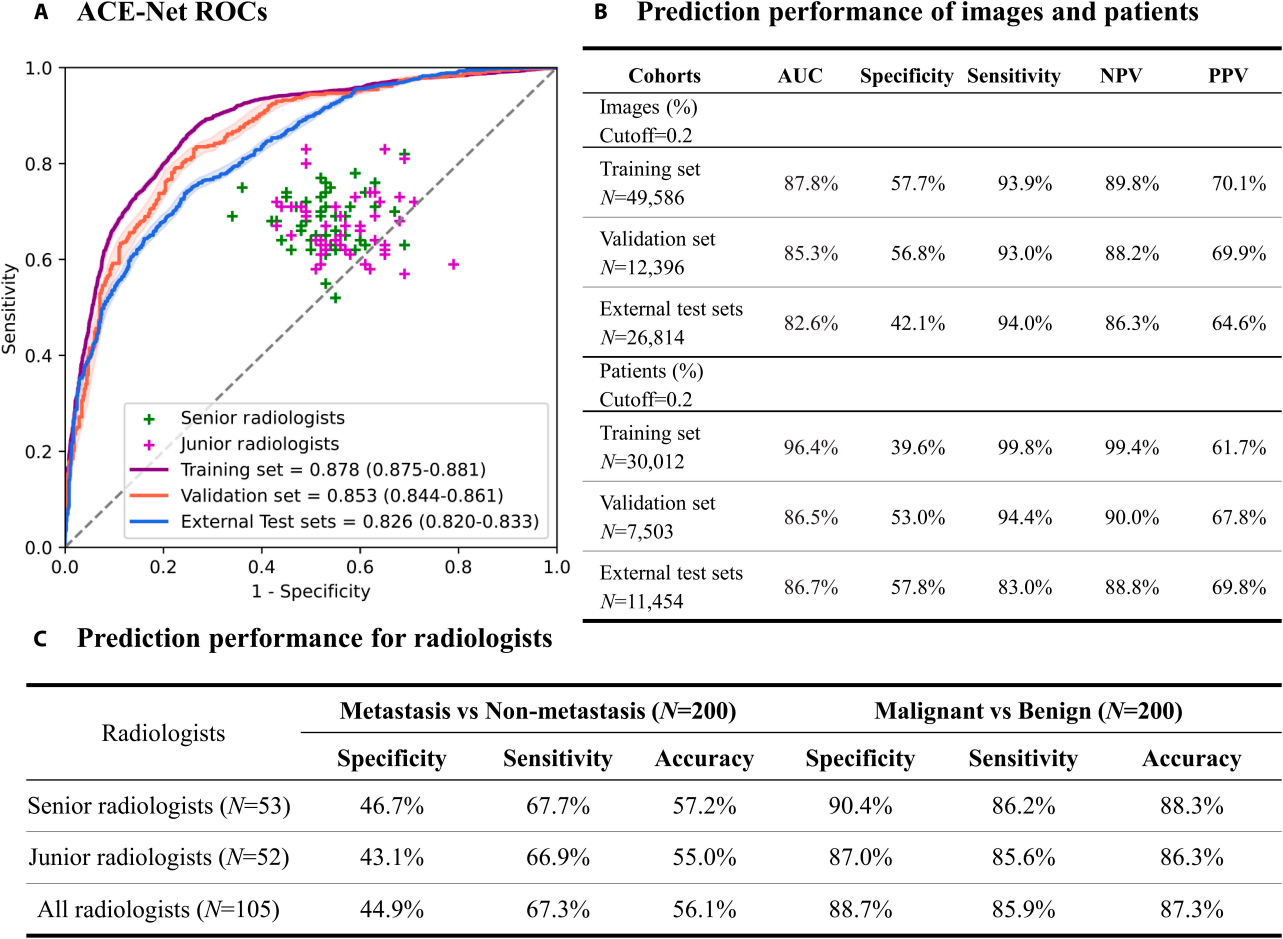
Prediction performance of models. (A) ACE-Net ROCs on the training set, validation set, and external test sets. (B) The table displays the specificity, sensitivity, AUC, NPV, and PPV prediction performance of images and patients across the 3 datasets. (C) Prediction performance of radiologists on CLNM, malignant, and benign discrimination tests. They performed the diagnosis of thyroid cancer with a high accuracy (0.873) but were extremely low (0.561) in CLNM prediction.

Figure 3 illustrates the performance of ACE-Net and human experts in their respective predictions of CLNM. ACE-Net exhibits noteworthy performance, with AUC values of 0.878 (CI: 0.875 to 0.881) on the training dataset and 0.826 (CI: 0.820 to 0.833) on the external test dataset. These results underscore its exceptional capacity for generalization. The predictive accuracy of human experts is 0.561 on the external test dataset. We conducted comparative experiments by benchmarking it against other previously published AI methods, which can be found in Table S1. These findings suggest that direct human expert predictions exhibit comparatively lower discriminatory performance when compared to ACE-Net. The model’s high precision in terms of sensitivity and NPV ensures its guiding significance for lymph node dissection surgery in Fig. 3B.

Furthermore, as depicted in Fig. 3C, our study involved the participation of 105 clinicians, comprising 53 senior radiologists (with over 3 years of clinical experience) and 52 junior radiologists (with less than 3 years of clinical experience). It is noteworthy that these radiologists demonstrated a high level of accuracy in thyroid cancer diagnosis, achieving an accuracy rate of 0.873, with a sensitivity of 0.859, and specificity of 0.887 when assessing a dataset comprising 100 benign and 100 malignant nodules. However, when it came to the classification accuracy of CLNM prediction, the results were notably low (accuracy = 0.561, sensitivity = 0.673, specificity = 0.449), Moreover, this low accuracy was consistent across both senior and junior radiologists, indicating a limited ability to predict CLNM based on conventional clinical knowledge and human expertise, suggesting that current clinical knowledge and human experience alone fall short of effectively predicting CLNM from ultrasound images.

### The standardized quantitative interpretation of clinical mechanisms

**Fig. 4.**
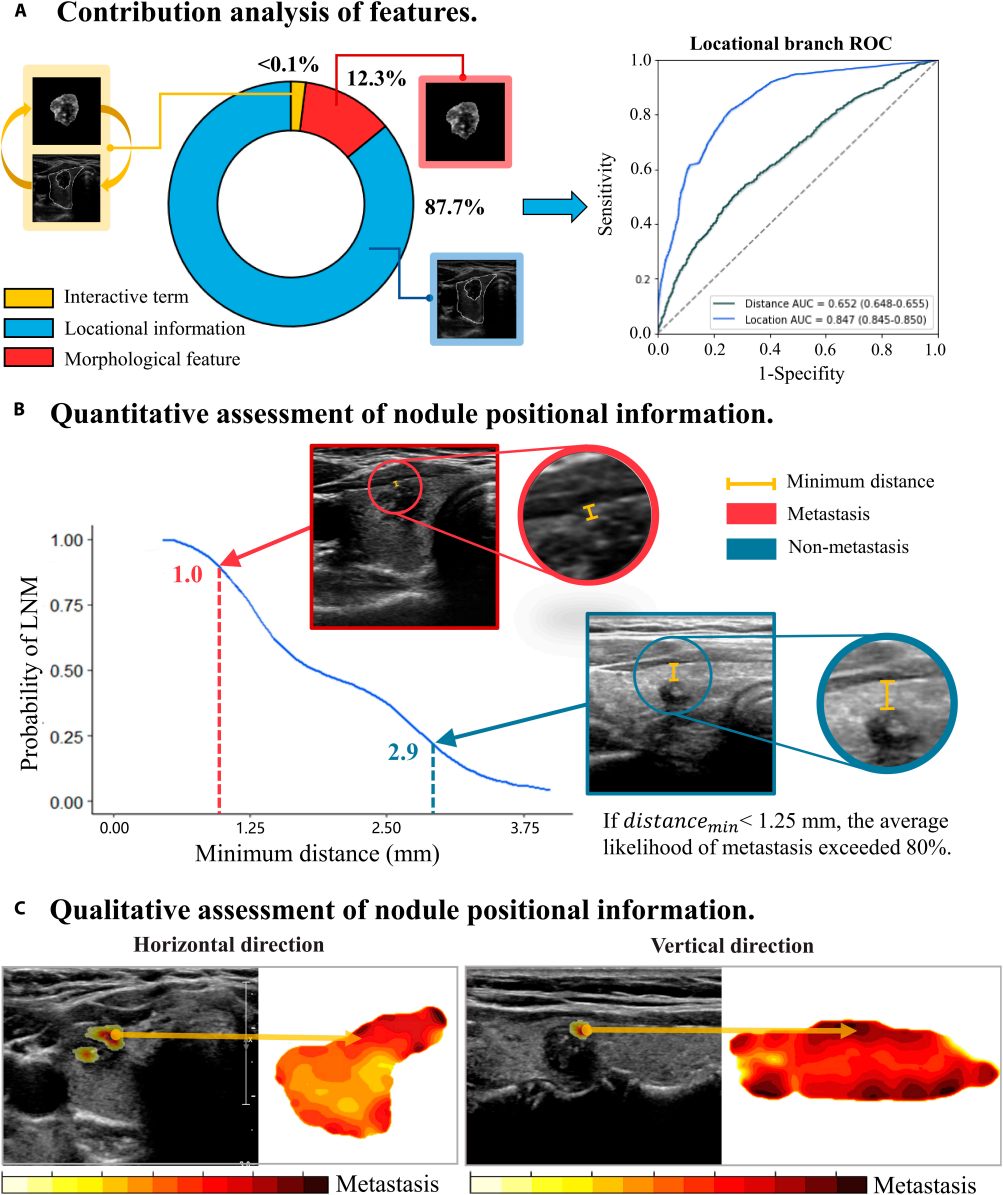
Qualitative and quantitative assessment of nodule positional information. (A) Through the application of ANOVA, we assessed the respective contributions of location information and morphological features to CLNM across the entire dataset. It is worth noting that the ROC analysis involving the minimum distance alone underscores its inadequacy as a standalone predictor of CLNM. (B) The curve depicted a gradual decrease in the probability of CLNM as the distance increased. Two examples were presented in the figure, one representing a metastasis image (outlined in red) and the other a nonmetastasis image (outline in green). The figure also illustrated the corresponding minimum distance between these images with a yellow line. (C) In the horizontal direction panel, the image displays a heat map, where the region corresponding to the higher color bar values indicates an elevated risk of CLNM, specifically pertaining to the horizontal thyroid orientation. On the left side of the heatmap, there is a sample of ultrasound images in black and white. Within the locational branch, the SAT extracts information from this example. The presence of red patches in the image indicates the attention areas of the graph network utilized in CLNM prediction. It is noteworthy that the likelihood of CLNM increases as a region’s proximity to a high-intensity area on the heat map becomes closer. In this particular example, the attention area is situated within a high-risk region, thus indicating a potential association with CLNM. In the vertical direction panel, the image portrays a thyroid in a vertical orientation, corresponding to the risk heat map on the right-hand side. On the left, there is an illustrative example related to CLNM.

Figure 4A illustrates the ANOVA analysis concerning the contribution of ability [morphology feature and chance (location information)] to CLNM prediction within ACE-Net. The findings reveal that morphological features accounted for 12.3% of the predictive capability (AUC = 0.628, CI: 0.624 to 0.631), while locational information constituted the predominant factor, contributing 87.7% to the prediction (AUC = 0.847, CI: 0.845 to 0.850).

To obtain both qualitative and quantitative insights into the locational information, we conducted a gradient analysis on the locational branch of ACE-Net. Subsequently, we identified the parameters with the most significant influence on the network and applied their weights to corresponding points on the thyroid nodules. These influential points were found to be near the edge of the thyroid capsule, and we designated them as “transition points”. Notably, when the minimum distance between the nodule edge around these “transition points” and the thyroid capsule was less than 1.25 mm, the average likelihood of metastasis exceeded 80% (Fig. 4B). However, it is important to note that the AUC of the prediction model based solely on the minimum distance was only 0.652 (Fig. 4A). Consequently, we hypothesized that, in addition to the distance factor, the risk of CLNM was also correlated with the specific location of the nodules within the thyroid. Through the consolidation of the data gathered from “transition points”, we created heatmaps illustrating the risk of CLNM based on nodule location (Fig. 4C) Our analysis revealed that regions near the thyroid capsule and the medial isthmus were characterized by a high risk of CLNM, with the lower thyroid region demonstrating a greater susceptibility to CLNM compare to the upper region. These heatmaps serve as valuable supplements for assessing CLNM risk and underscore that ACE-Net offers a more comprehensive approach than human experts who rely on subjective and localized assessments for predictions.

### Model evaluation of high-risk patients for metastasis

In our cohort, all patients underwent CLN dissection, a substantial portion of which was unnecessary. Given our CLNM prediction model, we proposed a model-based strategy that only conducted the CLN dissection for patients whose predicted CLNM risk was higher than a given threshold. The decision threshold should further be decided according to the cost–benefit ratio in specific clinical setting.

**Fig. 5.**
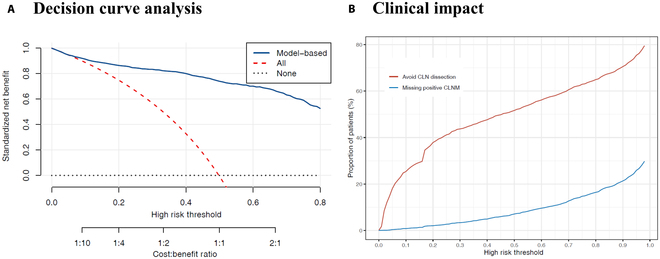
Decision curve analysis and clinical impact. (A) Decision curve analysis: the standardized net benefit (The benefit of effective CLN dissection − The cost of unnecessary CLN dissection) of the model-based strategy—the strategy that does CLN dissection for “All patients” and “None patients”. (B) Clinical impact of the model-based strategy. Red line: Proportion of patients that avoid CLN dissection under model-based strategy. Blue line: Proportion of missing postive CLNM cases.

The decision curve analysis (Fig. 5A) shows the net clinical benefit (benefit of effective CLN dissection − cost of unnecessary CLN dissection) of our model-based strategy. Along various cost–benefit ratio settings, our model-based strategy is superior to naïve strategies that conduct CLN dissection for all or none patients.

Figure 5B shows the clinical impact of the model-based strategy in our cohort. Under a risk threshold of 0.2, the model-based strategy could greatly reduce the need for CLN dissection in 37.9% of patients, while resulting in only 2.0% of missed positive CLNM cases.

## Discussion

The necessity of prophylactic lymph node surgery during thyroidectomy remains contentious due to the absence of standardized assessment protocols. In this study, we introduced the Competency and Opportunity Assessment Network (ACE-Net), leveraging thyroid ultrasound images to predict CLNM. Furthermore, we rigorously validated ACE-Net through external multicenter testing nationwide. In a comparative evaluation between ACE-Net and a panel of 105 human experts, the latter showed high accuracy in distinguishing benign from malignant nodules. However, their predictive accuracy for CLNM based on ultrasound characteristics of nodules was relatively low (accuracy = 0.561). In contrast, ACE-Net, with an AUC of 0.826 (CI = 0.820 to 0.833), demonstrated superior capability in extracting and interpreting subtle features from ultrasound images—features often challenging for human experts to recognize and quantify. This enabled ACE-Net to outperform human assessments in predicting CLNM. The approach to intraoperative prophylactic clearance of central regional lymph nodes varies significantly across different countries. In regions where routine prophylactic dissection is endorsed by guidelines, our model substantially reduces unnecessary lymph node dissections by 37.9%, without missing positive cases. Conversely, in regions where routine prophylactic dissection is not recommended, ACE-Net can identify high-risk patients, suggesting lymph node dissection to diminish recurrence risks. This underscores ACE-Net’s potential as a significant clinical tool, optimizing therapeutic strategies and enhancing patient outcomes.

The occurrence of CLNM is contingent upon the migration of tumor cells through lymphatic vessels. Hence, 2 distinct categories of factors bear significant relevance: (a) the intrinsic biological characteristics of tumor cells, which dictate their propensity for invasion and metastasis, and (b) the specific location of the malignancy, which governs the likelihood of tumor cell migration through neighboring lymphatic vessels. In previous research endeavors, greater emphasis has been placed on evaluating the metastatic potential of tumor cells, primarily owing to its accessibility via genetic profiling. In contrast, investigations into the migratory potential of tumor cells have historically received less attention. To date, a well-quantified assessment of CLNM risk linked to thyroid node location remains elusive. This is partly attributable to the inherent challenges faced by human experts in quantifying and standardizing thyroid node location. In contrast to the prevailing body of prior AI investigations, our model not only demonstrates a superior predictive capability for CLNM compared to human experts but also furnishes a systematic framework for interpreting AI-generated predictions.

1. Our model comprises 2 interpretable branches, with location information contributing to 87.7% and morphological features to 12.3% of the predictive capacity. This distribution underscores the model’s heavy reliance on location information for its prediction. It is important to emphasize that our research provides quantitative evidence of an association between ultrasound features, encompassing both morphology and location, and the occurrence of CLNM in thyroid cancer cases. This association implies morphology and nodule location being key contributors to the manifestation of CLNM. The discerned association within the dataset holds clinical significance as it furnishes a valuable tool for predicting CLNM and aiding clinical decision-making.

2. We provided nodules with a standardized and precise 3-dimensional (3D)-coordinated description across diverse backgrounds through uniform positioning. In doing so, we unearthed that deep learning possesses the capability to extract crucial information pertinent to CLNM, surpassing the information content offered by commonly employed features such as “back membrane breakthrough” and “minimum distance”.

To elaborate on the influence of specific factors within the location information on prediction outcomes, it becomes imperative to automate the assessment of the location relationships and distributions of nodules across the entire population. Based on this, we designed a homogeneous positioning method, which includes a deep learning-based diffeomorphism registration technique that enables the transformation of nodule images originating from diverse patients and ultrasound devices into a standardized thyroid framework. It is worth emphasizing that this endeavor represents a highly intricate and demanding undertaking. Moreover, to comprehensively assess the risk of CLNM within the growth regions of diverse thyroid nodules, we made substantial adaptations to the SAT to be used as a framework to design the module structure for extracting location features within the homogeneous positioning method. These modifications enabled SAT to compute the significance of points situated along the perimeters of all nodules, thereby identifying the pivotal points crucial to prediction. Additionally, we introduced a novel approach wherein we visualize the mapping results of nodules and thyroid location information within a high-dimensional space as a heat map. This innovative visualization technique represents a pioneering advancement in the realm of medical imaging analysis.

The generated heatmap has been instrumental in the identification of high-risk regions associated with CLNM, thereby offering novel and quantifiable insights for clinical applications. Specifically, our findings highlight the heightened susceptibility of areas proximate to the thyroid capsule and the medical isthmus to lymph node metastasis from thyroid cancer. Furthermore, our analysis indicates that the lower thyroid region exhibits a greater predisposition to CLNM compared to the upper region. These identified high-risk regions may exhibit a robust correlation with lymph node drainage pathways. Historically, cancer treatments have predominantly centered on the intrinsic biological attributes of tumors. However, our study suggests an alternative and promising strategy in the management of various cancer types. By focusing on mitigating the likelihood of metastasis, such as through the strategic blockage of high-risk lymphomatous metastasis pathways, we can potentially revolutionize cancer management approaches. Furthermore, our ACE-Net model holds the potential to facilitate the identification of target regions and beneficiary populations. Through an in-depth analysis of the location branch parameters, we have discerned that certain key points play a pivotal role in the prediction and are closely situated near the thyroid capsule. We have coined these critical points as the “transition points” of thyroid nodules, as they serve as optimal indicators of location-related CLNM risk. Notably, we have achieved a groundbreaking milestone by quantifying, for the first time, the precise association between the risk of CLNM and the distance separating these transition points from the thyroid capsule. This empirical evidence confirms the distinct and unique contributions of transition points to various risk zones. Furthermore, when the minimum distance between the nodule edge around the “transition points” and the thyroid capsule measures less than 1.25 mm, we observed an average metastasis probability exceeding 80%. Hence, it becomes imperative to incorporate both the growth area and the distance of nodules when making predictions related to CLNM. In this context, ACE-Net demonstrates a notable advantage by yielding comprehensive results that encompass both qualitative and quantitative aspects, surpassing the judgments of human experts, which are often subjective and localized in nature.

For several years, AI models have demonstrated a formidable capacity to extract information from data. However, the inherent lack of interpretability has posed challenges in translating this extracted information into comprehensible knowledge for clinicians. In our research, we have pioneered an interpretable AI-based research methodology that empowers us to harness AI for the extraction of human-comprehensible features. This methodology facilitates the transformation of previously subjective and ambiguous clinical features into objectively measurable and precise quantitative indicators. Consequently, it allows us to furnish data-driven clinical knowledge grounded in empirical evidence. Importantly this research method holds significant promise for broad-scale application in future medical research endeavors. Furthermore, the predictive network trained for CLNM exhibits a high degree of applicability and generalizability. Notably, its validity has been substantiated through rigorous testing across multiple external centers and diverse subgroups. Our comprehensive study spanned a wide geographic scope, encompassing various regions across China, including north China, east China, and northeast China, among others. Importantly, our study population comprised individuals from numerous ethnic backgrounds, such as Han, Mongolian, Manchu, and Hui, enhancing the inclusivity and representativeness of our findings.

In conclusion, our AI model serves as a valuable prediction tool for CLNM in thyroid cancer, significantly enhancing clinical decision-making capabilities. We believe that the interpretable deep learning approach employed in our study holds immense potential for uncovering novel clinical insights from medical data.

### Conclusion

In this diagnostic study, we developed an interpretable deep learning model that can be implemented as an AI support system for CLNM risk assessment based on thyroid ultrasound images. This model provides qualitative and quantitative clinical explanations for prediction based on the homogeneous positioning method. AI support led to a significant increase in diagnostic performance of board-certified radiologists and radiology residents.

## Data Availability

The data utilized in this study are subject to privacy restrictions. However, upon a reasonable request to the corresponding author, the data can be anonymized and made accessible for further analysis. The code utilized in this study may be made available upon reasonable request to the corresponding author. We have published some of the data for the Human-AI Competing Test on website (https://github.com/Snowinbio/ACE-Net).

## Supplementary Materials

Supplementary 1 Sections S1 to S6 Tables S1 to S5 Fig. S1 References
