# Supplementary material for: Thyroid Cancer Central Lymph Node Metastasis Risk Stratification Based on Homogeneous Positioning Deep Learning
Source: Research (Wash D C). 2024 Aug 20;7:0432. doi: 10.34133/research.0432 (PMC11334714; doi:10.34133/research.0432)
Supplement: Supplementary 1 — Sections S1 to S6 Tables S1 to S5 Fig. S1 References [file research.0432.f1.docx]

### Supplemental Methods

#### Section S1

#### Homogeneous Positioning: Registration and Transform

To facilitate large-scale statistics analyses and enable the comparison of positional relationships between thyroid and nodules, accounting for variation in resolution and angles resulting from extrusion, magnification, and reduction during the ultrasonic detection process, it was imperative to standardize all images to a consistent scale. This standardization process involved the registration of segmented thyroid and nodule ultrasound images into a unified thyroid template. Utilizing the deformation field derived from the thyroid registration, each nodule was systematically mapped onto the thyroid template. This meticulous registration procedure facilitated the automatic extraction of position information from the registered image. We meticulously scrutinized all ultrasound images both before and after the registration process. While some individuals exhibited deviation in relative position before and after registration, it is noteworthy that the vast majority of ultrasound images maintained consistency in their position relationships when compared to the original image.

The registration process was executed using the symmetric diffeomorphism-based algorithm. This involved working with an atlas feature matrix denoted as $F_{l}$ and a target feature matrix represented as $F_{l}^{R}$, both expressed as functions $F_{l},F_{l}^{R}:R$. Algorithom^1-3^ postulates that the diffeomorphism $\varphi$ is defined within the feature matrix domain $\Omega$ , bridging these feature matrices in such a way that $F_{l}^{R}=F_{l}\cdot\varphi^{-1}$. The boundary point $\varphi=\phi_{1}$ of the curve $\varphi=\phi_{t}, t\in\left[ 0,1 \right]$ satisfies the ordinary differential equation (o.d.e.):

$\frac{d\phi_{t}}{dt}=v_{t}\left( \phi_{t} \right), \phi_{0}=Fd, t\in\left[ 0,1 \right]$ (1)

Here $\phi_{0}=Fd$ presents the identity transformation, and $v_{t}$ denotes the time-dependent, smooth velocity field defined as $v_{t}:\Omega\to R, t\in\left[ 0,1 \right]$. The calculation of $\varphi$ is carried out as follows: $\varphi=\phi_{1}=\int_{0}^{1} v_{t}\left( \phi_{t} \right)dt$ with $\phi_{0}=Fd$, where we estimate the optimal $v_{t}$ by solving the standard Large Deformation Diffeomorphic Metric Matching (LDDMM) equation^4^:

$\hat{v}=\underset{v:\frac{d\phi_{t}}{dt}=v_{t}\left( \phi_{t} \right)}{arg min} \left( \int_{0}^{1} \left| \left| L\nu_{t} \right| \right|_{L^{2}}^{2}dt+\frac{1}{\sigma^{2}}\left| \left| F_{l}\cdot\varphi^{-1}-F_{l}^{R} \right| \right|_{L^{2}}^{2} \right)$ (2)

Where $L$ is the smoothness operator defined by equation: $L=-\alpha\nabla^{2}+\gamma F$, where $\nabla^{2}$ is the Laplacian operator. We used linear interpolation for image transformation. Mutual information served as the optimization metric during the registration process, and the final evaluation index employed was the mean square error (MSE).

$MSE=\frac{1}{MN}\sum_{y=1}^{M} \sum_{x=1}^{N} \left[ F_{l}\left( x,y \right)-F_{l}^{R}\left( x,y \right) \right]^{2}$ (3)

where $M$ and $N$ represented the row and column dimension of the matrix respectively.

Besides, we defined the transformation $T$ to compute the distance between the thyroid and nodal points as edges, with points as nodes, transforming $F_{l}^{R}$ into network data $G_{F_{l}^{R}}$. Data $G_{F_{l}^{R}}$ would be transmitted to the subsequent feature extractor $E_{l}$.

#### Section S2

#### Structure-Aware Graph Transformer Module

The feature extractor $E_{l}$ consists of three components: $k$-hop subgraphs, GNN (Graph Neural Network) extractor, and Structure-Aware Transformer. For node $u$, the GNN extractor aggregates the updated node representations of the entire $k$-hop subgraph using a pooling function. We assume that $N_{k}\left( u \right)$ represents the $k$-hop neighborhood of node $u$ including itself. ${GNN}_{G_{F_{l}^{R}}}^{\left( k \right)}$ represents a GNN model with $k$ layers applied to the graph $G_{F_{l}^{R}}$. Therefore, the representation $\xi$ of a node $u$ is:

$\xi\left( u,G_{F_{l}^{R}} \right)=\sum_{v\in N_{k}\left( u \right)} {GNN}_{G_{F_{l}^{R}}}^{\left( k \right)}\left( v \right)$ (4)

In the Structure-Aware Transformer, following the self-attention function are a multi-hop connection, a feed-forward network (FFN), and normalization layers connected before and after the FFN. We defined $d_{v}$ as the degree of node $v$, and the structural formula is as follows:

$F_{v}^{'}=F_{v}+\frac{1}{\sqrt{d_{v}}}SAattn\left( v,\xi\right)$ (5)

Where $SAattn$ represents structure-aware attention. $F_{v}$ is the feature of node $v$, and $F_{v}^{'}$ is the new feature of node $v$ after being extracted by $E_{l}$.

#### Section S3

#### ACE-Net Development

Following the processes of segmentation and registration, the thyroid was successfully mapped to a stable position. Consequently, the precise physical position data of both the nodule and thyroid could be converted into relative position information of the nodule within the current registration image. The locational branch employed for predicting CLNM was structured as a graph structure aware transformer^5^. This architecture allowed for the establishment of location relationships in a higher-dimension space, enabling more efficient exploration of the inherent correspondence within each set of locational data. Hence, in the current study, we put forth a novel approach named homogeneous positioning method wherein the positional relationship between the thyroid and the nodules was initially converted into a graph-based structure. The distance between the nodules and the thyroid was subsequently defined by the edge weights within this graph. Secondly, we employed the structure-aware graph transformer to extract structural features from this mapping. Specifically, we meticulously evaluated the relationship between the overall position of the nodule and the thyroid.

Morphological characteristics serve as significant indicators of a nodule's malignancy, a factor closely associated with the likelihood of metastasis. In the morphological feature branch, our approach involves an initial step of normalizing the gray values within the segmented nodule image. Subsequently, we employ the Neighborhood Attention Transformer^6^ to extract both texture and morphological information from the nodule, enabling the prediction of CLNM.

Ultimately, the outcomes obtained from the morphological feature branch and the locational information branch are amalgamated to provide a comprehensive prediction of CLNM. The procedure steps are outlined as follows:

$logit\left( y \right)=\beta_{0}+\beta_{1}x_{1}+\beta_{2}x_{2}+\beta_{3}x_{1}x_{2}$ (6)

In the formula, $x_{1}$represents the predicted transition probability value based on morphological information, $x_{2}$ represents the predicted transition probability value based on locational information, and $y$ signifies the prediction output generated by this model. Within this predictive model, the logistic regression model was trained using the training set, while the verification set and test set were employed to assess its effectiveness. Moreover, the logistic method was applied, and its formulation remained consistent with the previously described approach. However, the fitting process incorporated data from all the training, validation, and test sets. Consequently, the significance of coefficients $\beta_{1}, \beta_{2}, and \beta_{3}$ within the model was duly considered. Please refer to the accompanying text chart for detailed results.

We employed ANOVA to calculate the contribution rate of the input data. Following the computation of the predicted probability, ANOVA was utilized to assess the contribution of morphological and locational information to the overall probability. ANOVA entails the segregation of sources of sample variance and testing of hypotheses. The definition of sample variance is expressed by the following equation:

$s^{2}=\frac{1}{n-1}\sum_{i=1}^{n} {(y_{i}-\bar{y})}^{2}$ (7)

In the equation, $s^{2}$ represents the variance, and n-1 denotes the number of degrees of freedom. ANOVA employs the F-test to compare the factors contributing to total deviation, which is the ratio of variance between treatments to variance within treatments, for the purpose of hypothesis testing.

The subsequent formula provides a composite assessment of a patient’s CLNM risk. Here $P_{i}(i=1\ldots n)$ corresponds to the model’s prediction score for all ultrasound images of the patient, while ${Score}_{patient}$ signifies the synthetic CLNM risk prediction score based on the model. Additionally, $\varepsilon$=0.001 is a small constant chosen to prevent cases of infinite values. Through logistic regression, the patient's probability of lymph node metastasis can be recalculated from ${Score}_{patient}$.

${Score}_{patient}=-\frac{1}{n}\sum_{i=1}^{n} log(1-p_{i}+\varepsilon)$ (8)

#### Section S4

#### AI Model Interpretation

We employed the analysis of variance (ANOVA)^7-9^ analysis method to compute the contribution ratio of various features towards the predictive effectiveness of the model. This calculation was based on the deviance values associated with different factors within the model. Additionally, we utilized the proportion of deviation to determine the contribution ratio, as depicted below:

$Contribution Ratio\left（ Model \right）=\frac{Deviance(Model)}{\sum Deviance}$ (9)

We also employed the locally estimated scatterplot smoothing (LOESS) regression method to achieve a smoother representation of the relationship between the minimal distance separating the nodule from the dorsal thyroid membrane and the predicted CLNM probability as determined by locational branch. To minimize random errors and compute the distance between each pixel on the nodule’s edge and the thyroid capsule, we calculated the average distance from the nearest 40 pixels to the capsule as the minimum pixel distance. Subsequently, this pixel distance was converted into a physical distance using the ultrasonic image’s scale.

In line with the prediction outcomes of the deep graph convolution neural network, we computed the parameter gradients and employed saliency maps^10^ to generate risk heat maps corresponding to the location of nodule growth within each region.

#### Section S5

#### Statistical Analysis

Negative Predictive Value (NPV) is the probability that a person testing negative for a disease truly does not have the disease. In other words, it’s the percentage of negative results that are correct. The formula for NPV is:

$NPV=\frac{True negative}{True negative+False negative}$ (10)

Positive Predictive Value (PPV) is the probability that a person testing positive for a disease truly has the disease. It’s the percentage of positive results that are correct. The formula for PPV is:

$PPV=\frac{True positive}{True positive+False positive}$ (11)

Sensitivity (also referred to as the true positive rate and the recall) is the proportion of positives that are correctly identified as follows:

$Sensitivity/TPR=\frac{True positive}{True positive+False negative}$ (12)

Specificity (also known as the true negative rate), which measures the proportion of correctly identified negatives, was calculated as follows:

$Specificity/TNR =\frac{True negative}{True negative+False positive}$ (13)

AUC, standing for Area under the ROC Curve, measuring the entire two-dimensional area underneath the entire ROC curve (think integral calculus) from (0,0) to (1,1), was calculated as:

$A=\int_{0}^{1} TPR(FPR^{-1}(x))dx$ (14)

Where $FPR=1-TNR=1-Specificity$.

Given two bounding boxes $b_{1}$ and $b_{2}$, their IoU could be computed as:

$IOU\left( b_{1},b_{2} \right)=\frac{A\left( b_{1}\cap b_{2} \right)}{A(b_{1}\cup b_{2})}=\frac{A\left( b_{1}\cap b_{2} \right)}{A\left( b_{1} \right)+A(b_{2})-A(b_{1}\cap b_{2})}$ (15)

Where A(·) was the area of the shape. The calculation of IoU could therefore be formulated as a problem involving the computation of the area of each spherical rectangle and the intersection of two spherical rectangles.

#### Section S6

**Hyperparameter Choices**

In the nodule segmentation module, We used the ResNet-50 for the backbone of optimization-based foreground network,43 which was pre-trained on Image-Net.44 We set sizes of the convolution kernel to 4 × 4, 8 × 8, 16 × 16, and 32 × 32. Additionally, the learning rate was set to 0.007, and the maximum training iterations were set to 60000. While training the U-Net network1, for the thyroid segmentation module, we set batch-size to 8, used the Adam optimizer, set the learning rate to 0.001, and set the maximum epoch to 300.

To train the NAT module, we converted the images into 3-channel images with dimensions of 224×224, set the batch size to 32, used AdamW as the optimizer with a learning rate of 0.001, set the warm-up epochs to 20, and employed Binary Cross-Entropy as the loss function. The training ran for 300 epochs. For the training of the SAT module, we set the batch size to 32, used the AdamW optimizer with a standard warm-up strategy, set the warm-up steps to 5000, and set the learning rate to 0.001. Cross-entropy loss was used as the loss function, and the training ran for 300 epochs.

### Supplemental Tables

Table S1 Performance on geographic locations, ultrasonic equipment and ethnicities in external test sets.

| **Performance** | **Patient number** | **AUC** |
| --- | --- | --- |
| **Performance on geographic locations** |  |  |
| Tianjin | 1682 | 93.4% |
| Shanghai | 2141 | 86.4% |
| Nanjing | 2497 | 88.2% |
| Dongyang | 1892 | 82.6% |
| Xuzhou | 1705 | 85.9% |
| Ulanhot | 1145 | 81.7% |
| Yiwu | 392 | 89.2% |
| **Performance on ultrasonic equipment** |  |  |
| PHILIPS HD11, PHILIPS HD15, PHILIPS IU22,  PHILIPS EPIQ5 | 3823 | 89.5% |
| Esaote MYLAB Class C, Esaote MYLAB 90 | 2497 | 88.2% |
| SAMSUNG RS80A | 1892 | 82.6% |
| MINDRAY MT1010 | 1705 | 85.9% |
| GE LOGOQ E9 | 1145 | 81.7% |
| ACUSON S2000 | 392 | 89.2% |
| **Performance on ethnicities** |  |  |
| Han | 9605 | 87.0% |
| Mongolians | 815 | 83.3% |
| Manchus | 563 | 85.9% |
| Huis | 471 | 87.8% |

Table S2 Nodule information for datasets.

| **Nodule Characteristics** | Training set  N=44370 | Validation set  N=10059 | Test set  N=19940 |
| --- | --- | --- | --- |
| Tumor size, mm |  |  |  |
| Median (Quartiles) | 7.1 (4.0-11.3) | 7.3 (4.1-11.3) | 8.5 (5.4-14.4) |
| Mean±SD | 9.4±7.4 | 9.5±7.1 | 10.8±8.2 |
| Metastatic tumor, No. |  |  |  |
| Metastatic | 22451 | 5082 | 10089 |
| Non-metastatic | 21919 | 4977 | 9851 |

Table S3 The performance of segmentation module.

| Data^a^ | Training set  N=4020 | Validation set  N=1005 | Test set  N=1005 |
| --- | --- | --- | --- |
| Thyroid Segmentation (mIoU) | 0.885 | 0.864 | 0.792 |
| Nodule Segmentation (mIoU) | 0.877 | 0.832 | 0.811 |

a. The table displays performance of segmentation at the image level, where "N" in the table represents the number of images annotated by the radiologists.

Table S4 AUC predictive performance of thyroid cancer subtypes.

| Thyroid Cancer Subtypes (%)^a^ | Training set | | | Validation set | External test sets |
| --- | --- | --- | --- | --- | --- |
| FTC | | 78.3%  N=2056 | 75.1%  N=512 | | 73.4%  N=834 |
| MTC | | 76.7%  N=482 | 69.5%  N=120 | | 70.8%  N=303 |

a. The table displays predictive results at the patient level, where "N" in the table represents the number of patients.

Table S5 Comparison of prediction performance among different AI methods on validation sets.

| **Method ^a^** | **Specificity** | **Sensitivity** | **Accuracy** | **AUC** |
| --- | --- | --- | --- | --- |
| Thy-Net^11^ | 66.6% | 72.7% | 69.7% | 72.4% |
| ResNet^12^ | 68.8% | 72.5% | 70.7% | 71.6% |
| Inception V3^13^ | 65.8% | 72.3% | 69.1% | 69.3% |
| DenseNet^14^ | 56.4% | 71.9% | 64.2% | 64.4% |
| KNN^15^ | 63.5% | 70.5% | 67.0% | 68.0% |
| 1D-CNN^16^ | 66.1% | 69.8% | 68.0% | 72.5% |
| **ACE-Net** | **78.0%** | **78.2%** | **78.1%** | **85.3%** |

a. All methods utilized the same data preprocessing, model tuning techniques, etc.

To illustrate the performance of our model, we conducted comparative experiments by benchmarking it against other previously published AI methods. The outcomes of these experiments on the external test set are presented in Table S1. The results have shown that our model achieves the highest predictive accuracy among currently published papers, while also providing clinical explanations not found in any previous research.

### Supplemental Figures


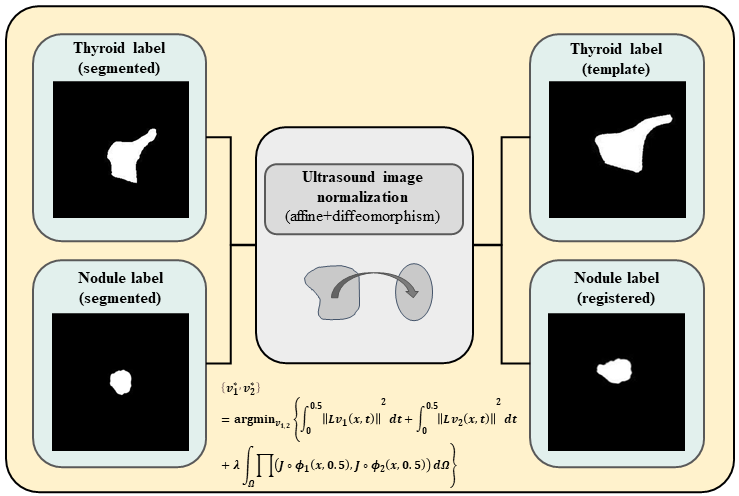


**Figure S1. Registration process flow chart.**

Through ultrasound image normalization, segmented thyroid label, thyroid label template, and segmented nodule label can obtain the registered nodule label (affine and diffeomorphism).

### Reference

1. Avants BB, Tustison N, Song G, others. Advanced normalization tools (ANTS). *Insight j*. 2009;2(365):1–35.

2. Lin L, Wang J, Fu Z, others. Multi-atlas segmentation of mouse brain MRM based on optimized advanced normalization tools. *Science Discovery Ayutyanont*. 2017;5(6):486–491.

3. Tustison NJ, Cook PA, Holbrook AJ, et al. The ANTsX ecosystem for quantitative biological and medical imaging. *Scientific reports*. 2021;11(1):1–13.

4. Glaunès J, Qiu A, Miller MI, Younes L. Large deformation diffeomorphic metric curve mapping. *International journal of computer vision*. 2008;80(3):317–336.

5. Chen D, O’Bray L, Borgwardt K. Structure-aware transformer for graph representation learning. PMLR; 2022:3469-3489.

6. Hassani A, Walton S, Li J, Li S, Shi H. Neighborhood attention transformer. 2023:6185-6194.

7. Anuradha D, Kalpanapriya D. Intuitionistic fuzzy ANOVA and its application in medical diagnosis. *Research Journal of Pharmacy and Technology*. 2018;11(2):653–656.

8. Gevrey M, Dimopoulos I, Lek S. Review and comparison of methods to study the contribution of variables in artificial neural network models. *Ecological modelling*. 2003;160(3):249–264.

9. Quinlan JR, others. Learning with continuous classes. 343–348.

10. Simonyan K, Vedaldi A, Zisserman A. Deep inside convolutional networks: Visualising image classification models and saliency maps. *arXiv preprint arXiv:13126034*. 2013;

11. Yao J, Lei Z, Yue W, et al. DeepThy‐Net: A Multimodal Deep Learning Method for Predicting Cervical Lymph Node Metastasis in Papillary Thyroid Cancer. *Advanced Intelligent Systems*. 2022;4(10)doi:10.1002/aisy.202200100

12. Lee JH, Ha EJ, Kim JH. Application of deep learning to the diagnosis of cervical lymph node metastasis from thyroid cancer with CT. *European Radiology*. 2019/10/01 2019;29(10):5452-5457. doi:10.1007/s00330-019-06098-8

13. Yu J, Deng Y, Liu T, et al. Lymph node metastasis prediction of papillary thyroid carcinoma based on transfer learning radiomics. *Nature Communications*. 2020/09/23 2020;11(1):4807. doi:10.1038/s41467-020-18497-3

14. Wang C, Yu P, Zhang H, et al. Artificial intelligence–based prediction of cervical lymph node metastasis in papillary thyroid cancer with CT. *European Radiology*. 2023/05/13 2023;doi:10.1007/s00330-023-09700-2

15. Qin H, Que Q, Lin P, et al. Magnetic resonance imaging (MRI) radiomics of papillary thyroid cancer (PTC): a comparison of predictive performance of multiple classifiers modeling to identify cervical lymph node metastases before surgery. *La radiologia medica*. 2021/10/01 2021;126(10):1312-1327. doi:10.1007/s11547-021-01393-1

16. Wang Z, Qu L, Chen Q, et al. Deep learning-based multifeature integration robustly predicts central lymph node metastasis in papillary thyroid cancer. *BMC Cancer*. 2023/02/08 2023;23(1):128. doi:10.1186/s12885-023-10598-8
